# Longitudinal efficacy and toxicity of SARS-CoV-2 vaccination in cancer patients treated with immunotherapy

**DOI:** 10.1038/s41419-022-05548-4

**Published:** 2023-01-20

**Authors:** Pavlina Spiliopoulou, Helena J. Janse van Rensburg, Lisa Avery, Vathany Kulasingam, Albiruni Razak, Philippe Bedard, Aaron Hansen, Andrzej Chruscinski, Ben Wang, Maria Kulikova, Rachel Chen, Vanessa Speers, Alisa Nguyen, Jasmine Lee, Bryan Coburn, Anna Spreafico, Lillian L. Siu

**Affiliations:** 1 Princess Margaret Cancer Centre, University Health Network, Toronto, ON Canada; 2 Department of Internal Medicine, University of Toronto, Toronto, ON Canada; 3 Dalla Lana School of Public Health, University of Toronto, Toronto, ON Canada; 4 Laboratory Medicine Program, University Health Network, Toronto, ON Canada; 5 Mutli-Organ Transplant Program, University Health Network, Toronto, ON Canada

**Keywords:** Translational research, Vaccines

## Abstract

Despite more than 2 years having elapsed since the onset of SARS-CoV-2 pandemic, a level of hesitation around increased SARS-CoV-2 vaccine toxicity in cancer patients receiving immunotherapy (IO) remains. This hesitation stems from the idea that IO agents could elicit an overwhelming immune stimulation post vaccination and therefore increase the risk of vaccine-related toxicity. The aim of our study was to explore serological responses to SARS-CoV-2 vaccination in patients treated with IO and describe the level of immune stimulation using parameters such as blood cytokines, autoantibody levels and immune related adverse events (irAEs) post vaccination. Fifty-one evaluable patients were enrolled in this longitudinal study. Absolute levels and neutralization potential of anti-SARS-CoV-2 antibodies were not significantly different in the IO group compared to non-IO. Chemotherapy adversely affected seroconversion when compared to IO and/or targeted treatment. Following vaccination, the prevalence of grade ≥2 irAEs in patients treated with IO was not higher than the usual reported IO toxicity. We report, for the first time, that anti-SARS-CoV-2 vaccination, elicited the generation of five autoantibodies. The significantly increased autoantibodies were IgM autoantibodies against beta-2 glycoprotein (*p* = 0.02), myeloperoxidase (*p* = 0.03), nucleosome (*p* = 0.041), SPLUNC2 (*p* < 0.001) and IgG autoantibody against Myosin Heavy Chain 6 (MYH6) (*p* < 0.001). Overall, comprehensive analysis of a small cohort showed that co-administration of SARS-CoV-2 vaccine and IO is not associated with increased irAEs. Nevertheless, the detection of autoantibodies post anti-SARS-CoV-2 vaccination warrants further investigation (NCT03702309).

## Background

For more than 2 years, the world has faced tremendous public health, economic, and ethical challenges due to the Severe Acute Respiratory Syndrome Coronavirus 2 (SARS-CoV-2) pandemic [1]. Cancer patients have comprised a particularly vulnerable population during this time. When infected with SARS-CoV-2, patients with cancer have higher rates of morbidity and mortality than their non-cancer peers [2, 3]. Interruptions in anti-cancer treatments due to SARS-CoV-2 infection have also led to worse oncological outcomes [4].

The development of vaccines preventing severe disease, hospitalization and death from COVID-19 has marked a significant achievement [5, 6]. Despite their immense contribution to the path towards resolution of this pandemic, antibody responses and antibody neutralizing potential wane over the months following SARS-CoV-2 primary infection or immunization [7, 8]. SARS-CoV-2 variants with novel spike protein mutations enable escape from host antibody responses for both vaccinated and convalescent people [9–11] and pose a major obstacle. So far, five variants have been nominated as variants of concern (VOC) and have stood out for their heightened infectivity; these are namely variants B.1.1.7 (Alpha), B.1.351 (Beta), P.1 (Gamma), B.1.617.2 (Delta), and more recently, B.1.1.529 (Omicron) with several sub-variants currently being active.

Cancer patients have been prioritized for receipt of initial vaccination series and boosters around the globe. Despite this, there is ongoing concern as to whether vaccine efficacy is impacted by increased immune vulnerability in the setting of malignancy or secondary to immune-modulating therapies (such as immuno-oncology (IO) agents). The heterogeneous nature of neoplastic diseases and diverse therapies applied has further complicated efforts to interrogate this relationship.

Emerging evidence suggests that the use of certain anti-cancer therapies during SARS-CoV-2 vaccination may alter immune correlates of protection. In some cancer patients, including a high proportion of those receiving cytotoxic or B-cell depleting therapies, lower rates of seropositivity and T-cell responses have been reported after two doses of mRNA vaccine [12–15]. Vaccine efficacy in patients treated with immune checkpoint inhibitors (ICIs) appears to be comparable to that observed in healthy individuals, although questions surrounding an altered risk of immune related adverse events (irAEs) after vaccination have been posed [16–18]. In particular, the possibility of aberrant cytokine production with elevated IL-2, IL-6, CXCL8, CCL2, and sIL-1R, after SARS-CoV-2 vaccination in patients receiving ICIs has only recently emerged in preliminary reports [19, 20]. Further, while autoantibody development has been correlated with irAEs in patients receiving IO treatment, whether SARS-CoV-2 vaccination can affect autoantibody generation is entirely unknown [21, 22]. With vaccination programmes ongoing, questions regarding SARS-CoV-2 vaccination in cancer patients receiving IO agents warrant urgent exploration.

In the present study, we comprehensively explored peripheral immune responses to SARS-CoV-2 vaccination in IO-treated cancer patients. We also described the immune-mediated toxicities in IO-treated patients who received anti-SARS-CoV-2 vaccination and lastly, we conducted an in-depth analysis of auto-antibody generation following vaccination.

## Methods

### Patient samples and data collection

Patients with a diagnosis of solid malignancy, treated at Princess Margaret Cancer Centre, University Health Network (PM-UHN), with either a standard of care (SOC) therapy or within a clinical trial were recruited. Blood samples from patients enroled were banked under the study *Immune Response to COVID-19 Vaccine in immunotherapy (IO) and non-IO Treated Cancer Patients* (VIVACIOUS, NCT03702309) between March 2021 and July 2022. Patients were allowed to have any of the approved vaccines in Canada at the time of study recruitment, such as Pfizer (BNT162b2), Moderna (mRNA-1273), AstraZeneca [AZD1222 (ChAdOx1)] or their approved combinations.

Sampling at 6 time points per patient were undertaken: at baseline (BASE), between 1st and 2nd vaccine doses (PRE2D), at 1 week (1 W), 1 month (1 M), 4–6 months (4–6 M) and 10–12 months (10–12 M) after the 2nd dose, as per Fig. S1A. Sampling between 1st and 2nd vaccine dose had no particular fixed timeline, as long as it was performed > 10 days after the first vaccination dose. At each time point, 1 × 10 mL serum separator tube (SST) was collected and spun at 2000 g for 10 min at 4 ^o^C. After processing, serum samples were aliquoted and stored in liquid nitrogen vapor phase for long term storage until time of downstream analysis.

Patient demographics, cancer diagnosis, past medical history, treatment details, concomitant medications, and immune related adverse events (irAEs) of ≥grade 2 severity by Common Terminology Criteria for Adverse events Version 5.0 (CTCAE 5.0) were collected prospectively and entered into a Medidata Rave database. This de-identified database is password protected and encrypted, to be compliant with PM-UHN standards for all investigator-initiated studies.

The most updated Charlson Co-morbidity Index (CCI) was used to describe patients’ overall health status [23]. CCI is a method of predicting mortality by weighting scores to common comorbidities and has been widely utilized by health researchers to measure burden of disease. The total CCI score attained correlates with a specific 10-year survival rate with total score of 1 having a 96% rate of 10-year survival and any score higher than 3 correlating with a 0% rate of 10-year survival. Most non-malignant comorbidities score 1 point and every decade of life after the age of 50 adds an extra point. Localised cancer scores 2 points and metastatic cancer scores 6 points.

This study was conducted with the approval of UHN Oncology Research Ethics Board (CAPCR/UHN REB #: 21–5797) and all patients signed informed consent. Participants who were concurrently treated in an interventional study with experimental treatment, were allowed to take part in this observational study (VIVACIOUS), according to their respective study protocols; or were not specifically disallowed to do so, if protocols were silent on this matter. The laboratory exploratory analysis was performed by scientific staff who was blinded to patients’ identity.

### Antibodies

Serum samples from patients (20 µL) were analyzed using Elecsys® Anti-SARS-CoV-2 S assay on the cobas e411 analyzer (Roche Diagnostics) for the quantitative detection of total antibodies (IgM/IgG/IgA) to the SARS-CoV-2 spike (S) protein receptor binding domain (RBD). This assay is a double-antigen sandwich electrochemiluminescence immunoassay, which uses streptavidin-coated microparticles to separate bound from unbound substances prior to applying a voltage to the electrode. Application of a voltage to the electrode then induces chemiluminescent emission which is measured by a photomultiplier. Samples were run in singleton. The measuring range of this assay is 0.40–250 U/mL (up to 2500 U/mL with on-board 1:10 dilution), with a concentration of ≥0.80 U/mL considered positive. Serum antibody levels against SARS-CoV-2 S protein are the result of immune response to either natural infection or vaccination but cannot distinguish between the two [24].

### Surrogate virus neutralization test (sVNT)

The GenScript SARS-CoV-2 surrogate Virus Neutralization Test (sVNT) Kit was used to detect circulating neutralizing antibodies against SARS-CoV-2 that block the interaction between the viral S glycoprotein RBD with the angiotensin converting enzyme-2 (ACE2) cell surface receptor. The interaction between RBD and ACE2 leads to viral endocytosis into the host cells and subsequent viral replication. This assay detects any antibodies in serum and/or plasma that neutralize the RBD-ACE2 interaction.

Horseradish peroxidase conjugated RBD was incubated with 150 µL of diluted patient serum sample and incubated at 37 ^o^C (30 min) with respective positive and negative controls. Samples were then loaded on plate wells and incubated at 37 ^o^C for 15 min. Absorbance was measured at 450 nm. A result of ≥30% is considered positive for the detection of SARS-CoV-2 neutralizing antibodies [25].

sVNT assay measures the potential of serum antibodies to block viral entry and provides an additional level of information over and above what the quantitative measurement of antibody levels provides. The presence of neutralizing antibodies has directly been correlated with better outcomes in subjects with COVID19 infection, whereas low levels are linked with higher likelihood of re-infection [26, 27].

### Cytokine measurement

Frozen serum samples were used for the measurement of cytokine concentrations using a bead-based multiplex assay (Human Th1/Th2 Magnetic Luminex Performance Assay 11-plex, R&D Systems, Canada) and the Bio-Plex 200 Systems (Bio-Rad, Canada), according to the manufacturer’s protocol. The analytes detected with this assay are GM-CSF, IFN-γ, IL-1β/IL-1F2, IL-2, IL-4, IL-5, IL-6, IL-10, IL-12 p70, IL-13, and TNF-α. Samples were diluted 1:2. Standard curves were used to calculate plasma cytokine concentration using Bioplex manager software (version 5.0, Bio-Rad, United States).

### Autoantibodies

Recent evidence has focused on the sensitivity of autoantibodies to identify patient risk of developing irAEs when treated with cancer immunotherapy and mainly, immune checkpoint inhibitors [28, 29]. Our group has previously utilised and validated a customized auto-antibody panel with the ability to detect a wide range of both IgM and IgG antibodies against 161 self-antigens [30, 31]. The antigen library used for the arrays is shown in Supplemental Table 1 (table S1). Briefly, 161 antigens were spotted in duplicate onto 2-pad FAST nitrocellulose slides (GVS, Sanford, ME, USA) using a robotic microarrayer (Virtek, Waterloo, Canada) outfitted with solid pins (Arrayit Corporation, Sunnyvale, CA, USA). Slides were blocked overnight in blocking buffer (PBS 0.1% Tween and 5% FCS). The slides were then probed with patient serum (diluted 1:100) in blocking buffer for 1 h at 4 °C. After washing, the slides were probed with a pair of fluorescently labelled anti-human secondary antibodies (Cy3-labelled goat anti-human IgG Fc and Alexa Fluor 647-labelled goat anti-human IgM; Jackson ImmunoResearch, West Grove, PA, USA) for 45 min at 4 °C in blocking buffer. After additional washing, the slides were dried by centrifugation for 5 min. The slides were scanned with an Axon 4200 A scanner (Molecular Devices, Sunnyvale, CA, USA) in an ozone free enclosure, and fluorescent intensities were quantified using Genepix software Version 6.1 (Molecular Devices, Sunnyvale, CA, USA).

### Data analysis

#### Study design

We assumed that ~30% of patients treated with IO (monotherapy or in combination) experience grade 2/3 immune related adverse events. With this assumption, we calculated that with a cohort of IO patients of *n* = 37, our study will have 84% power to detect an increase in the rate of irAEs from 30% to 50% with a type-I error rate of 6.0%. Recruitment of IO and non-IO patients was simultaneous, and we halted recruitment when our IO cohort was close to reaching *n* = 37 (ultimately *n* = 35 patients treated with IO). As patient recruitment progressed, booster doses of vaccination gradually became approved. This change introduced significant heterogeneity in our population of patients, and we concluded that further accrual will significantly contaminate our results.

#### Antibody levels

Longitudinal plots were used to illustrate the changes in antibody levels over time and number of vaccinations received for patients on IO vs non-IO therapy. Univariate regression analyses were used to determine if antibody levels one-month post vaccination were associated with age, sex, IO therapy, chemotherapy, number of doses received, time since first dose and vaccine type(s). For this analysis spike antibody levels were log-transformed to normalise the distribution and Holm’s correction was applied to *p*-values to control for multiple comparisons. Differences in antibody levels at 4 months between those who received two vs. three vaccinations were evaluated with Wilcoxon rank sum tests.

#### Cytokine levels

Cytokine levels under the limit of detection were recoded to 0. Changes in cytokine levels were examined using longitudinal plots and Wilcoxon signed rank test for paired data between baseline and the assessments between doses and 1 week after the second vaccination.

#### Auto-antibodies

Autoantibody profiling in serum using antigen microarrays was performed as described previously [30, 32]. We used this panel to investigate for signals of autoimmunity precipitated by vaccination in the IO-treated group (*n* = 33), with a small number of randomly selected non-IO treated patient samples (*n* = 5), as control. Baseline (pre) and following second vaccination samples (post) were compared. All “post” samples were collected at 1 week (1 W) following second vaccination, apart from five samples that were collected at 1 month (1 M) following second vaccination. Log-transformation was applied to normalise the distributions and mixed effects ANOVA models were fit to each antibody to investigate the between-sub effect of therapy group (IO vs non-IO) and the within subject effect of time (pre-post vaccination). In addition, a Time x Therapy interaction was modelled to determine if the changes over time were different between the treatment groups. A holm-adjustment was applied to the resulting *p*-values (of which there were three per antibody). An exploratory analysis was conducted to determine if any relationships existed between cytokine levels and the autoantibodies that were significantly elevated following vaccination. Spearman rank correlations were assessed and relationships significant after controlling for multiple corrections using a Holm correction were identified. R was used for all statistical analyses.

## Results

### Patient characteristics

Fifty-three patients were enroled in this study and 51 patients were evaluable (defined as having received at least one dose of SARS-CoV-2 vaccination, Fig. S1B), with a median follow-up of 335 days (range, 40–453). The number of patients receiving 3rd and 4th vaccine doses reduced with time either due to lower vaccine uptake or due to cancer-associated mortality. All 51 patients received at least 2 doses of vaccination, 43 patients (84%) received at least three doses, and 21 (41%) patients received a 4^th^ dose. Patients were separated into those treated with an immunotherapeutic compound or a combination containing at least one immunotherapy agent (IO, *n* = 35) and those treated with cytotoxic/cytostatic agents with no immunostimulatory properties (non-IO, *n* = 16), during their vaccine series. Patients’ demographics and other characteristics at baseline are shown in Table 1. One patient switched treatment from IO (immune checkpoint inhibitor, ICI) to non-IO (chemotherapy) between their first and second vaccine dose and was therefore included in the non-IO group of patients. All the analyses described in this manuscript were also performed by excluding this patient (data not shown). As the statistical significance of our results did not change between the two analyses, we concluded that this patient should be included in the non-IO cohort.

**Table 1:** Patients characteristics at baseline.

	IO	Non-IO	Total
	*n* = 35	*n* = 16	*n* = 51
Sex			
female	14 (40%)	10 (63%)	24 (47%)
male	21 (60%)	6 (37%)	27 (53%)
Age			
median (min–max)	65 (26–78)	62 (47–85)	64 (26–85)
Cancer type			
head & neck	8 (23%)	7 (44%)	15 (29%)
melanoma	14 (40%)	0 (0%)	14 (27%)
breast	0 (0%)	6 (37%)	6 (12%)
GI—gastroesophageal	2 (6%)	2 (13%)	4 (8%)
GI—colorectal	3 (9%)	0 (0%)	3 (6%)
GI—HPB	3 (9%)	0 (0%)	3 (6%)
gynaecological	3 (9%)	0 (0%)	3 (6%)
sarcoma	2 (6%)	1 (6%)	3 (6%)
Cancer stage			
M1	30 (86%)	13 (81%)	43 (84%)
M0	5 (14%)	3 (19%)	8 (16%)
Charlson comorbidity index (CCI)			
median (min–max)	8 (4–12)	8 (4–10)	8 (4–12)
Treatment during primary vaccination series			
clinical trial	26 (74%)	7 (44%)	33 (65%)
SOC	9 (26%)	9 (56%)	18 (35%)
Type of treatment			
ICI	16 (46%)	-	16 (31%)
ICI + targeted	7 (20%)	-	7 (14%)
ICI + investigational IO	6 (17%)	-	6 (12%)
Investigational IO	4 (11%)	-	4 (8%)
ICI + chemotherapy	2 (6%)	-	2 (4%)
targeted	-	12 (75%)	12 (24%)
chemotherapy	-	4 (25%)	4 (8%)
Vaccine (primary series)			
BNT162b2 (Pfizer)	24 (69%)	10 (63%)	34 (67%)
mRNA-1273 (Moderna)	6 (17%)	3 (19%)	9 (18%)
BNT162b2/mRNA-1273	3 (9%)	1 (6%)	4 (8%)
AZD1222 (AstraZeneca)	1 (3%)	2 (13%)	3 (6%)
AZD1222/BNT162b2	1 (3%)	0 (0%)	1 (2%)
SARS-Cov-2 infection			
pre-vaccination	0 (0%)	0 (0%)	0 (0%)
post-vaccination	4 (11%)	2 (13%)	6 (12%)
Booster vaccines			
3rd dose	29 (83%)	14 (86%)	43 (84%)
4th dose	12 (34%)	9 (56%)	21 (41%)

Abbreviations = *IO* immunotherapy, *GI* gastrointestinal, *HPB* hepatobiliary, *SOC* standard of care, *ICI* immune checkpoint inhibitor, *AZ* AstraZeneca.

Most patients recruited had head and neck cancer (29%), followed by melanoma (27%) and breast cancer (12%). Most patients had metastatic disease (84%) and the median CCI score for all patients was 8 [4–12]. A score of 8 points confers a 0% rate of 10-year survival. Patients in the IO group mostly received ICI based treatments; 46% ICI treatment alone, 20% ICI plus targeted treatment, 17% ICI plus a novel immunotherapeutic, 11% a novel immunotherapeutic alone and 6% of patients received ICI plus chemotherapy. Patients in the non-IO group mostly received targeted treatments (75%) and only 25% of patients in this group were treated with chemotherapy. The composition of anti-cancer systemic treatments in this study reflects the inclusion of patients treated in a large Phase 1 clinical trials unit where patients had already exhausted standard of care therapies and were mainly recruited in studies investigating novel compounds and combinations (clinical trial 65% and standard of care 35%). In keeping with Canadian public health recommendations during the year 2021, the majority of patients received mRNA-based vaccines: BNT162b2 (Pfizer) = 67%, mRNA-1273 (Moderna) = 18% and BNT162b2/mRNA-1273 combination = 8%, whereas only 6% of the patients received viral vector-based vaccination with AZD1222 (AstraZeneca). None of the patients recruited in the study had been infected with SARS-CoV-2 before initial vaccination series, but 12% of patients contracted SARS-CoV-2 during their follow-up and after vaccination with the initial series.

### Antibody levels and neutralizing potential

**Fig. 1:**
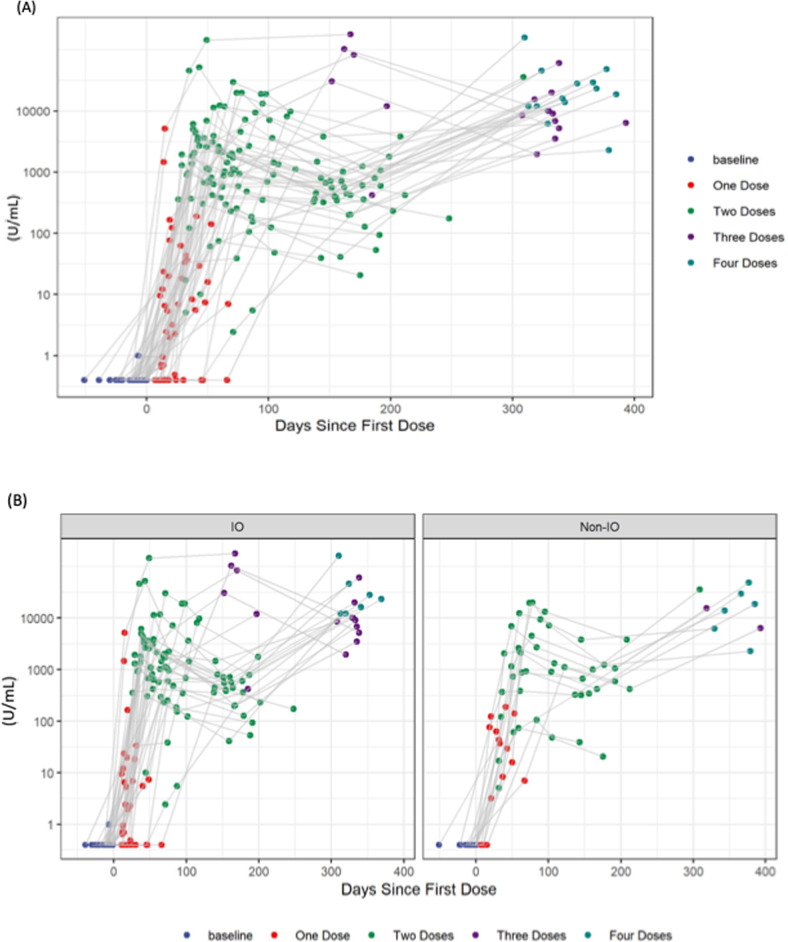
Antibody level dynamics over time. **A** Anti-RBD antibody levels for all patients in relation to time; colours indicate numbers of vaccine doses (1 dose: red; 2 doses: green; 3 doses: purple, 4 doses: light blue). **B** anti-RBD antibody levels for all patients in relation to time stratified into those treated with immunotherapy (IO) and non-immunotherapy (non-IO).

Anti-SARS-CoV-2 S-protein RBD antibody levels and neutralizing antibody levels were measured at six timepoints, as per study schema in Fig. S1A. Dynamics of both antibody concentration and neutralizing potential are similar between IO and non-IO groups, supporting the hypothesis that immunotherapy treatment does not alter anti-SARS-CoV-2 S-protein RBD antibody generation compared with non-IO treatment (Fig. 1A, B). More specifically, at 1 month (1 M) following two vaccination 2 doses, all participants had received no <2 vaccination doses. Median concentration of antibodies for the IO group was not statistically different from the non-IO group [median 1438 U/mL (Q1, Q3; 483.7–3107 U/mL) for IO group *vs* 1108 U/mL (Q1, Q3; 627.3–3609.5 U/mL) for non-IO group, *p* > 0.99]. Similarly, the neutralizing antibody level at 1 M was 94.5% (Q1, Q3; 85.9–95.4%) for the IO group and 92.7% (Q1, Q3; 80.8–95.1%, *p* > 0.99) for the non-IO group (Fig. S2A,B). At 1 M, the only factor that was significantly associated with antibody levels was sex, with male patients demonstrating both lower antibody levels and lower neutralizing potential than females (table S2). Six patients treated with chemotherapy had lower antibody levels, but this difference was not significant after adjusting for multiple comparisons.

**Fig. 2:**
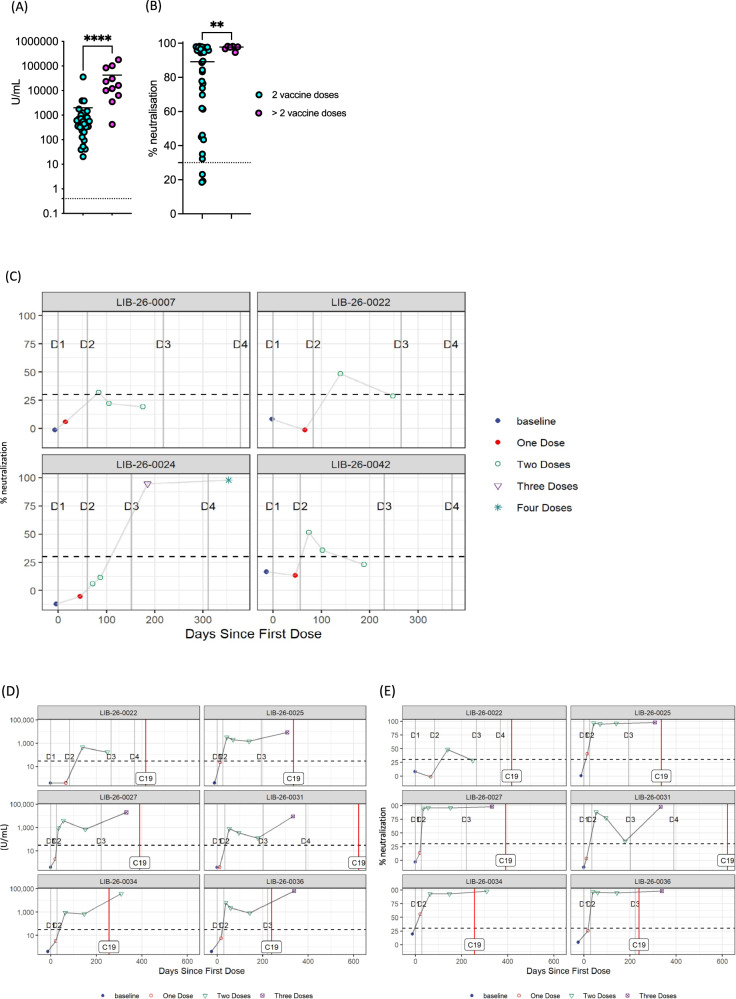
Antibody/neutralization levels after booster vaccination and special patient cases. **A** Anti-RBD antibody levels between patients received 2 vaccine doses (cyan, *n* = 29) versus patients received >2 vaccine doses (magenta, *n* = 11) at 4–6 months following completion of initial vaccination series (2 doses). **B** Neutralizing antibody levels between patients received 2 vaccine doses (cyan, *n* = 34) versus patients received >2 vaccine doses (magenta, *n* = 6) at 4–6 months following completion of initial vaccination series (2 doses). **C** Neutralizing antibody levels in patients with low neutralization in relation to time and administration of vaccination doses (D1: 1st dose, D2: 2nd dose, D3: 3rd dose and D4: 4th dose). **D** Anti-RBD antibody levels in patients who contracted SARS-CoV-2 in relation to vaccination doses and SARS-CoV-2 infection (shown as red line labelled C19). **E** Neutralizing antibody levels in patients who contracted SARS-CoV-2 in relation to vaccination doses and SARS-CoV-2 infection (shown as red line labelled C19). **A**, **B** Mann–Whitney test used for comparison between groups. (****p < 0.0001; **p < 0.01;).

Antibody levels waned over the next 10–12 months following first dose of the initial vaccination series, based on subsequent vaccine dosing. Notably, levels of both antibody concentration and neutralization begin to decrease swiftly after the second dose of the initial vaccination series and before further booster dose (Fig. S2C), except for one patient who developed COVID19 infection at 228 days following the second dose and has not received any subsequent doses. Despite the clear decline of antibodies over time following the second dose, subsequent booster doses are successful in inducing antibody generation anew. At 4–6 months following initial vaccination, patients with three vaccine doses had a higher median concentration (12030 U/mL *vs* 555.2 U/mL, *p* < 0.001) and neutralization levels (97.7 *vs* 89.1 *p* = 0.003) compared to patients who received only two vaccine doses (Fig. 2A,B), highlighting the importance of repeat vaccination to enhance humoral immunity amongst patients with cancer.

There were 4 patients with notably low neutralization and antibody concentration in this study (Fig. 2C). These patients were all male, with a median age of 69.5 years and 3/4 had the diagnosis of head and neck cancer. One of the head and neck cancer patients (LIB-26–0007) started chemotherapy between first and second dose of the first series and had low antibody concentration which marginally surpassed the threshold of neutralization (105.2 U/mL and 33%), 1 W after completion of the second dose. This level decreased further to a concentration of 48.5 U/mL and 22% neutralization within 1 month, following the second dose. This patient missed further blood collections and therefore, it is unknown whether subsequent vaccine doses had any impact on these low antibody levels. The other two patients with head and neck cancer, patients LIB-26–0042 and LIB-26–0022, had no clearly identifiable causes for their diminished antibody response; patient LIB-26–0022 had developed IO-related cutaneous lupus and was receiving topical corticosteroids; the patient had a remote history of receiving hydroxychloroquine for cutaneous lupus, but was not receiving systemic immunosuppressants at the time of their vaccination. The remaining patient with a primary tumor other than head and neck (LIB-26–0024), was treated with IO for skin cancer and had concomitant chronic lymphocytic leukaemia, for which treatment was not required. Interestingly, we observed extremely low levels of antibody concentration and absent neutralization in this patient (5.5 U/mL and 11.5%) at 1 M following second dose, which might be explained by the concomitant haematological malignancy. However, both antibody and neutralization levels were significantly improved after the patient received booster vaccination; antibody concentration of 421 U/mL and neutralization of 94.6% following 1 booster and antibody concentration of 28130 U/mL and neutralization of 97.9% after the second booster dose.

Lastly, six patients contracted COVID19 infection after vaccination (Fig. 2D and Fig. 2E). All of these infections occurred during Omicron variant wave (between 23/Dec/2021 and 31/May/2022). None of these patients was hospitalised or developed COVID19-related complications and 3/6 were asymptomatic. In at least 4 out of 6 patients, antibody concentration and neutralization were thought to be adequate at the time of infection (patients LIB-26–0025, LIB-26–0027, LIB-26–0034 and LIB-26–0036), while for the remaining 2 patients, samples immediately prior to their infection were not available. Despite the absence of a booster vaccine, patient LIB-26–0034 had high levels of antibody concentration and neutralization and was asymptomatic when they contracted SARS-CoV-2.

### Cytokines

**Fig. 3:**
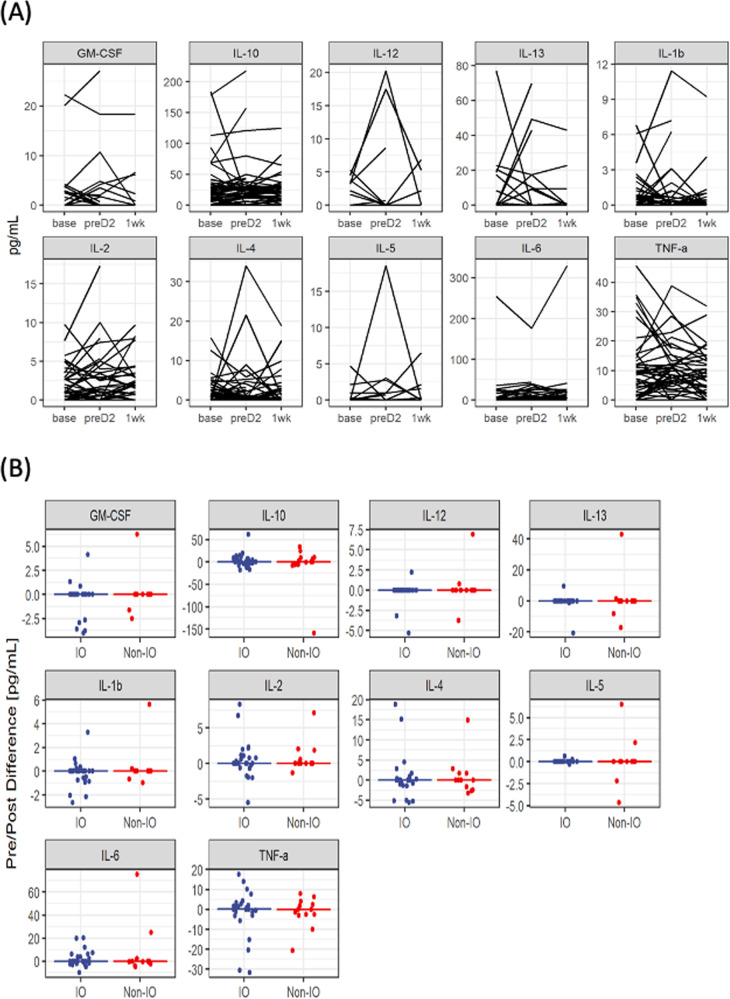
Cytokine dynamics during initial vaccination series. **A** Longitudinal plots of cytokine concentration (pg/mL) at baseline (BASE, *n* = 51), prior to second vaccination dose (preD2, *n* = 48), and 1 week (1wk, *n* = 42) after the second dose for all patients; lines connect paired samples, there were no statistically significant differences found by Wilcoxon matched pairs signed rank test. IFN-γ not shown as levels were undetectable at all timepoints. **B** Plots of change from baseline to 1 week post 2nd vaccination dose, comparing IO (*n* = 35)/non-IO (*n* = 16) treatment groups. Each point represents the change in median cytokine level from baseline to 1 week post second vaccine dose. The line represents the median of each group. There were no statistically significant differences found.

Cytokine levels were assessed at baseline, between the 2 initial series vaccine doses, and 1 week (1 W) after the second dose. Most cytokines (IL-5, IL-12, IL-13, GM-CSF and IFN-γ) were undetectable for most patients. Overall, there were no statistically significant differences between either the baseline and the intra-dose measurement, nor the baseline and post 1 week measurement (Fig. 3A). Figure 3B indicates that the lack of change in cytokine levels at 1 W timepoint compared to baseline is similar for the IO and non-IO groups.

### irAEs and auto-antibodies

Patients treated with IO were prospectively followed-up for irAEs of more than grade 2 severity, from the date of first vaccine dose to 90 days following the 4th dose. Two out of 35 (5.7%) patients experienced immune related toxicity after 1st dose and before the 2nd vaccine dose, 8/34 (23.5%) after the 2nd dose and before the 3rd, 2/29 (7%) following the 3rd dose and 2/13 (15.3%) after the 4th dose (Fig. S3). Most of these adverse events were characteristic of treatment with IO compounds (Table S3) with only neck lymphadenopathy being more rarely observed with IO and potentially induced by the vaccination. Overall, we did not observe an irAE prevalence higher than historical rates seen in clinical practice.

**Fig. 4:**
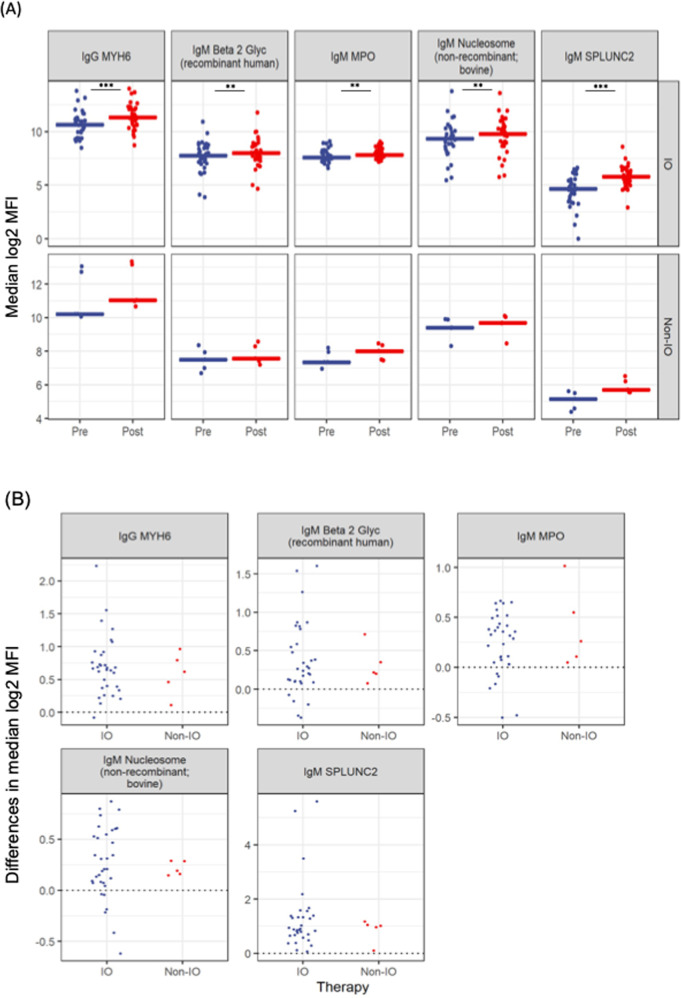
Auto-antibody dynamic changes before and after vaccination. **A** Comparisons between autoantibodies found to be statistically different between baseline (pre) and following second vaccination dose (post) as measured by median log2 fluorescence intensity (MFI) with a 161 self-antigen array. Top row shows patients treated with immunotherapy (IO, *n* = 33) and bottom row shows patients treated with non-immunotherapy (non-IO, *n* = 5). The logarithmic values of MFI are plotted here. Reported *p*-values indicate the effect of Time from the mixed effects ANOVA and refer to all patients (IO and non-IO) (***p < 0.001; **p < 0.01). There were no significant treatment effects. **B** Comparison of differences in MFI of the five autoantibodies of interest between IO (*n* = 33) and non-IO (*n* = 5) groups. All comparisons were statistically non-significant with mixed effects ANOVA.

We discovered 5 autoantibodies that were significantly elevated (after controlling for multiple comparisons) following vaccination, as defined by a significant increase of their mean log-transformed fluorescent intensity (mean Log2 MFI). These autoantibodies were an IgG antibody against Myosin heavy-chain 6 (MYH6) (mean Log2 MFI 11.5 vs 10.8, *p* < 0.001) and also IgM antibodies against: Human beta-2 glycoprotein 1 (mean Log2 MFI 8 vs 7.6, *p* = 0.02), Myeloperoxidase (MPO) (mean Log2 MFI 8 vs 7.7, *p* = 0.01), Nucleosome (median Log2 MFI 9.6 vs 9.3, *p* = 0.0413), and against Short Palate, Lung and Nasal epithelium carcinoma associated protein 2 (SPLUNC2) (mean Log2 MFI 5.8 vs 4.6, *p* < 0.001) (Fig. 4A). There were no significant effects of Treatment group, nor Time x Treatment group interactions, suggesting that IO treatment did not specifically contribute to the autoantibody elevation following vaccination (Fig. 4B). In view of the established connection between mRNA-based vaccination and development of myocarditis, we sought to investigate whether the increase of anti-MYH6 was linked to the type of vaccine received by looking for differences between the cohorts of patients received mRNA vaccination at both doses versus those who received non-mRNA vaccination at one of the two doses. We did not detect any differences, however, we urge caution here, as the sample size in the non-mRNA vaccine groups was extremely small (mean difference 0.7 *vs* 0.5, *p* = 0.60, Fig. S4). A moderate positive correlation (Spearman correlation = 0.61, adjusted *p* = 0.012) between IgM anti-MPO autoantibody and IL-2 cytokine levels post-vaccination was found (Fig. S5).

## Discussion

We conducted the VIVACIOUS study in a Canadian tertiary cancer centre during the height of initial SARS-CoV-2 pandemic waves. We aimed to understand multi-dimensional longitudinal immune correlates after anti-SARS-CoV-2 vaccination in solid cancer patients treated with immunotherapies and to compare these with patients receiving non-IO-based treatments. At that time, there was considerable vaccine hesitancy for some patients receiving cancer immunotherapies owing to emerging data on potentially autoimmune-related complications linked to mRNA vaccines (such as myocarditis). Thus, we further endeavoured to undertake an in-depth analysis of immunological and clinical parameters that could provide insights into this risk. Unlike other large series, we did this by adding multiple timepoint blood collections through the initial series and the booster vaccines doses and cytokine/autoantibody assays to assess this risk in a multi-dimensional manner.

In performing this analysis, we found that IO-treated cancer patients were largely indistinguishable from non-IO-treated cancer patients with respect to absolute levels and neutralization capability of anti-RBD antibody generation after primary series anti-SARS-CoV-2 vaccination. Although others have reported reduced rates of seroconversion after vaccination amongst patients with haematologic malignancies, we confirm that patients with solid cancer receiving immunotherapy generate robust antibody responses. Both quantitative and functional testing of anti-SaRS-CoV-2 antibodies following vaccination seem to be largely unaffected by cancer immunotherapy, providing surrogate evidence that B cell function remains intact during treatment with immunotherapy. This finding is in keeping with what is reported in the literature so far [14, 18, 33, 34] and provides reassurance that patients can achieve adequate anti-viral immunity despite receiving treatment that interferes with pivotal axes of the immune system. In contrast to immunotherapies, we found that chemotherapy adversely suppresses humoral immunity against SARS-CoV-2, when compared to non-myelotoxic regimens, a finding that has been validated by analyses of even larger patient cohorts [12–14, 16, 33].

As with healthy subjects, we further observed that serological responses wane over the 4–6 months following the second vaccine dose which highlights the importance of implementing booster vaccinations [7, 8]. A recent report by Ehmsen *et al*. showed that third and fourth vaccine doses can restore waning antibody titres in solid cancer patients [35]. We note that several studies have shown that higher titres of neutralizing antibodies correlate with reduced risk of symptomatic infection [36, 37]. Interestingly, Fendler *et al*. recently reported that 3 vaccination doses are imperative for achieving neutralizing antibodies that offer protection against the Omicron variant in patients with solid malignancies [38]. In our study, most patients infected with SARS-CoV-2 were affected during the Omicron wave, had recent proof of high levels of neutralization before being infected, and they largely experienced mild infection with no need for hospitalization.

Although most of our participants generated robust antibody responses after initial primary vaccine series, we identified several patients with low seroconversion. Interestingly, all of these patients were male and 3/4 of them had a head and neck primary. In the literature, there is conflicting data as to whether patient sex or primary tumor type have an impact on seroconversion after vaccination. At least one publication reported higher antibody titres amongst female solid cancer patients, an observation also made in our cohort of patients [39]. In our study, at least one patient was able to generate high antibody titres and viral neutralization capacity after third and fourth doses, indicating a potential utility for booster doses in patients with sub-optimal humoral responses after initial vaccine series.

We next examined whether cytokine production differed after vaccination in IO-treated and non-IO-treated patients. Given the broad immunomodulatory impacts of vaccination and immunotherapies, we were somewhat surprised to find no significant, lasting differences after vaccination in any of the cytokines studied. A moderate positive correlation was observed between IL-2 and anti-MPO autoantibody generation and interestingly, a recent case report on cytokine release syndrome following anti-SARS-CoV-2 vaccination in a patient receiving ICI was accompanied by an increase in IL-2 [19].

Having examined the impact of IO therapies on vaccine efficacy, we further explored whether vaccine administration might affect immunotherapy-related toxicities. Isolated thrombocytopenia (without thrombosis) has been described with mRNA-based COVID-19 vaccines [40] and a pathogenic platelet factor-4 (PF4)-dependent syndrome leading to thrombotic thrombocytopenia after vaccination with ChAdOx1 nCov-19 [41]. Moreover, myocarditis and pericarditis have been observed in younger patients undergoing vaccination with mRNA-based vaccine [42]. Our clinical observation of IO-treated patients did not show evidence of severe toxicity enhancement following anti-SARS-CoV-2 vaccination. irAEs reported were in both number and severity, in line with the clinically observed toxicity of immune checkpoint inhibitors. There were no observed cases of myocarditis or pericarditis in our cohort. Our clinical and immunological (cytokines, autoantibody array) findings are in concordance with other reports suggesting that anti-SARS-CoV-2 vaccination is safe for patients treated with immunotherapy [17, 18]. This will hopefully contribute to the growing evidence and support the development of vaccine trust amongst cancer patients.

Lastly, we observed an increase of five antibodies against self-antigens after vaccination, an effect that was not treatment dependent. Human beta-2 glycoprotein 1, a critical regulator of complement and coagulation systems, is also a target of anti-phospholipid antibodies implicated in the antiphospholipid syndrome [43]. In our study, it was found to be increased without a clear clinical correlate. Borghi et al. have reported that vaccination may trigger low titre autoantibody production, including anti-phospholipid antibodies, in health care workers [44]. In contrast, a recent study looking into the dynamics of auto-antibodies after SARS-CoV-2 vaccination in patients with antiphospholipid syndrome, did not observe a statistically significant increase of beta-2 glycoprotein 1 [45]. Moreover, we found an increase in antibodies against nucleosome, which is the cardinal antigen implicated in the pathogenesis of systemic lupus erythematosus [46] and an increase in antibodies against SPLUNC2 which belongs to an extended group of proteins expressed in the upper airways, nose and mouth, for which little is known [47].

Intriguingly, we observed an elevation of autoantibody against alpha-cardiac myosin heavy chain (MYH6), a component of the contractile system in cardiac muscle [48]. MYH6 has previously been shown to function as an autoantigen in myocarditis [49], and autoantibodies against MYH6 have been observed in patients hospitalized with COVID-19 [50]. Anand et al., recently reported that a variant of MYH6 has strong amino acid homology with the SARS-CoV-2 replicase polyprotein 1a/1ab [51]. Although vaccination with mRNA particles encoding for the S glycoprotein (which is not a replicase) could not directly explain this autoantibody elevation, this finding is intriguing and warrants further investigation. We show for the first time that this autoantibody is derepressed followed anti- SARS-CoV-2 vaccination and this finding can form the hypothesis of further mechanistic investigation into MYH6 protein biology. This could potentially reveal the pathophysiology of myocarditis linked to both natural infection and vaccination against SARS-CoV-2. Nonetheless, in our series, the increase in these five autoantibodies, did not correlate with clinical findings.

Our study has various limitations, such as the small number of patients included and the lack of characterising immune responses at a cellular level by interrogating IL-2/IFN-γ-expressing T lymphocyte responses and functionality. Moreover, for our neutralization test, we considered 30% as the threshold for positivity, although we note that more recent evidence has suggested that higher neutralization levels are required for adequate protection against infection [37]. In designing our study protocol, we attempted to select timepoints for sample collection that would allow for serological responses to develop, however in doing so, it is possible that we have missed biologically significant cytokine changes that are transient. Finally, while we have performed a multi-dimensional analysis of potential relationships between SARS-CoV-2 vaccination and IO therapy, we have not looked for the possibility that vaccine administration impacts anti-neoplastic therapy effectiveness in terms of cancer outcomes.

## Conclusion

In conclusion, we have found that administration of anti-SARS-CoV-2 vaccines can be safely delivered in patients undergoing cancer immunotherapy, without elevating the risk of immune related adverse events. The absolute and neutralizing levels of anti-RBD antibodies are decreasing with time and repeat booster vaccination is required to achieve desirable levels of immunity. The observation that anti-SARS-CoV-2 vaccination de-represses generation of five autoantibodies, a cardiac myosin amongst them, warrants further investigation.

## Supplementary information

Supplementary Tables and Figures

## Data Availability

Patient data is stored in a secure password protected network drive only available to study team. Data available upon request.

## References

[1] Gorbalenya AE, Baker SC, Baric RS, de Groot RJ, Drosten C, Gulyaeva AA (2020). The species severe acute respiratory syndrome-related coronavirus: classifying 2019-nCoV and naming it SARS-CoV-2. Nat Microbiol.

[2] Bakouny Z, Hawley JE, Choueiri TK, Peters S, Rini BI, Warner JL (2020). COVID-19 and cancer: current challenges and perspectives. Cancer Cell.

[3] Rüthrich MM, Giessen-Jung C, Borgmann S, Classen AY, Dolff S, Grüner B (2021). COVID-19 in cancer patients: clinical characteristics and outcome—an analysis of the LEOSS registry. Ann Hematol.

[4] Pinato DJ, Tabernero J, Bower M, Scotti L, Patel M, Colomba E (2021). Prevalence and impact of COVID-19 sequelae on treatment and survival of patients with cancer who recovered from SARS-CoV-2 infection: evidence from the OnCovid retrospective, multicentre registry study. Lancet Oncol.

[5] Baden LR, El Sahly HM, Essink B, Kotloff K, Frey S, Novak R (2020). Efficacy and safety of the mRNA-1273 SARS-CoV-2 vaccine. N. Engl J Med.

[6] Polack FP, Thomas SJ, Kitchin N, Absalon J, Gurtman A, Lockhart S (2020). Safety and efficacy of the BNT162b2 mRNA Covid-19 vaccine. N. Engl J Med.

[7] Naaber P, Tserel L, Kangro K, Sepp E, Jürjenson V, Adamson A (2021). Dynamics of antibody response to BNT162b2 vaccine after six months: a longitudinal prospective study. Lancet Regional Health - Eur.

[8] Israel A, Shenhar Y, Green I, Merzon E, Golan-Cohen A, Schäffer AA, et al. Large-scale study of antibody titer decay following BNT162b2 mRNA vaccine or SARS-CoV-2 infection. medRxiv. 2021. 10.1101/2021.08.19.21262111. 10.3390/vaccines10010064 PMC8781423 35062724

[9] Planas D, Veyer D, Baidaliuk A, Staropoli I, Guivel-Benhassine F, Rajah MM (2021). Reduced sensitivity of SARS-CoV-2 variant Delta to antibody neutralization. Nature.

[10] Bates TA, Leier HC, Lyski ZL, McBride SK, Coulter FJ, Weinstein JB (2021). Neutralization of SARS-CoV-2 variants by convalescent and BNT162b2 vaccinated serum. Nat Commun.

[11] Shen X, Tang H, Pajon R, Smith G, Glenn GM, Shi W (2021). Neutralization of SARS-CoV-2 variants B.1.429 and B.1.351. N. Engl J Med.

[12] Peeters M, Verbruggen L, Teuwen L, Vanhoutte G, Vande Kerckhove S, Peeters B, et al. Reduced humoral immune response after BNT162b2 coronavirus disease 2019 messenger RNA vaccination in cancer patients under antineoplastic treatment. ESMO Open. 2021;6:100274. 10.1016/j.esmoop.2021.100274 PMC8423808 34597941

[13] Oosting SF, van der Veldt AAM, GeurtsvanKessel CH, Fehrmann RSN, van Binnendijk RS, Dingemans A-MC (2021). mRNA-1273 COVID-19 vaccination in patients receiving chemotherapy, immunotherapy, or chemoimmunotherapy for solid tumours: a prospective, multicentre, non-inferiority trial. Lancet Oncol.

[14] Addeo A, Shah PK, Bordry N, Hudson RD, Albracht B, Di Marco M (2021). Immunogenicity of SARS-CoV-2 messenger RNA vaccines in patients with cancer. Cancer cell.

[15] Monin L, Laing AG, Muñoz-Ruiz M, McKenzie DR, del Molino del Barrio I, Alaguthurai T (2021). Safety and immunogenicity of one versus two doses of the COVID-19 vaccine BNT162b2 for patients with cancer: interim analysis of a prospective observational study. Lancet Oncol.

[16] Buttiron Webber T, Provinciali N, Musso M, Ugolini M, Boitano M, Clavarezza M (2021). Predictors of poor seroconversion and adverse events to SARS-CoV-2 mRNA BNT162b2 vaccine in cancer patients on active treatment. Eur J Cancer.

[17] Waissengrin B, Agbarya A, Safadi E, Padova H, Wolf I (2021). Short-term safety of the BNT162b2 mRNA COVID-19 vaccine in patients with cancer treated with immune checkpoint inhibitors. Lancet Oncol.

[18] Lasagna A, Agustoni F, Percivalle E, Borgetto S, Paulet A, Comolli G, et al. A snapshot of the immunogenicity, efficacy and safety of a full course of BNT162b2 anti-SARS-CoV-2 vaccine in cancer patients treated with PD-1/PD-L1 inhibitors: a longitudinal cohort study. ESMO Open. 2021;6:100272. 10.1016/j.esmoop.2021.100272 PMC8407964 34543863

[19] Au L, Fendler A, Shepherd STC, Rzeniewicz K, Cerrone M, Byrne F (2021). Cytokine release syndrome in a patient with colorectal cancer after vaccination with BNT162b2. Nat Med.

[20] Walle T, Bajaj S, Kraske JA, Rösner T, Cussigh CS, Kälber KA, et al. Cytokine release syndrome-like serum responses after COVID-19 vaccination are frequent but clinically inapparent in cancer patients under immune checkpoint therapy. medRxiv. 2021;3:1039–51. 10.1038/s43018-022-00398-7 PMC9499865 35715501

[21] Badran YR, Shih A, Leet D, Mooradian MJ, Coromilas A, Chen J (2020). Immune checkpoint inhibitor-associated celiac disease. J Immunother Cancer.

[22] Stamatouli AM, Quandt Z, Perdigoto AL, Clark PL, Kluger H, Weiss SA (2018). Collateral damage: insulin-dependent diabetes induced with checkpoint inhibitors. Diabetes.

[23] Quan H, Li B, Couris CM, Fushimi K, Graham P, Hider P (2011). Updating and validating the charlson comorbidity index and score for risk adjustment in hospital discharge abstracts using data from 6 countries. Am J Epidemiol.

[24] Burbelo PD, Riedo FX, Morishima C, Rawlings S, Smith D, Das S, et al. Detection of nucleocapsid antibody to SARS-CoV-2 is more sensitive than antibody to spike protein in COVID-19 patients. medRxiv. 2020. 10.1101/2020.04.20.20071423.

[25] Tan CW, Chia WN, Qin X, Liu P, Chen MI, Tiu C (2020). A SARS-CoV-2 surrogate virus neutralization test based on antibody-mediated blockage of ACE2-spike protein-protein interaction. Nat Biotechnol.

[26] Bergwerk M, Gonen T, Lustig Y, Amit S, Lipsitch M, Cohen C (2021). Covid-19 breakthrough infections in vaccinated health care workers. N. Engl J Med.

[27] Zhu F, Althaus T, Tan CW, Costantini A, Chia WN (2022). Van Vinh Chau N, et al. WHO international standard for SARS-CoV-2 antibodies to determine markers of protection. Lancet Microbe.

[28] Tang H, Geng R, Xu X, Wang Y, Zhou J, Zhang S, et al. Safety and efficacy of PD-1/PD-L1 inhibitors in cancer patients with preexisting autoantibodies. Front Immunology. 2022;13:893179. 10.3389/fimmu.2022.893179 PMC9148956 35651612

[29] Les I, Pérez-Francisco I, Cabero M, Sánchez C, Hidalgo M, Teijeira L, et al. Prediction of immune-related adverse events induced by immune checkpoint inhibitors with a panel of autoantibodies: protocol of a multicenter, prospective, observational cohort study. Front Pharmacol. 2022;13:894550. 10.3389/fphar.2022.894550 PMC9198493 35721217

[30] Chruscinski A, Huang FY, Nguyen A, Lioe J, Tumiati LC, Kozuszko S (2016). Generation of antigen microarrays to screen for autoantibodies in heart failure and heart transplantation. PLoS One.

[31] Genta S, Keshavarzi S, Yee N, Heirali A, Hansen AR, Siu LL (2022). Customized autoantibodies (autoAbs) profiling to predict and monitor immune-related adverse events (irAEs) in patients receiving immune checkpoint inhibitors (ICI). J Clin Oncol.

[32] Chruscinski A, Huang FYY, Ulndreaj A, Chua C, Fehlings M, Rao V, et al. Generation of two-color antigen microarrays for the simultaneous detection of IgG and IgM autoantibodies. J Vis Exp. 2016;15:54543. 10.3791/54543 PMC5092032 27685156

[33] Thakkar A, Gonzalez-Lugo JD, Goradia N, Gali R, Shapiro LC, Pradhan K (2021). Seroconversion rates following COVID-19 vaccination among patients with cancer. Cancer Cell.

[34] Figueiredo JC, Merin NM, Hamid O, Choi SY, Lemos T, Cozen W (2021). Longitudinal SARS-CoV-2 mRNA vaccine-induced humoral immune responses in patients with cancer. Cancer Res.

[35] Ehmsen S, Asmussen A, Jeppesen SS, Nilsson AC, Kragh A, Frederiksen H (2022). Increased antibody titers and reduced seronegativity following fourth mRNA COVID-19 vaccination in patients with cancer. Cancer Cell.

[36] Cromer D, Steain M, Reynaldi A, Schlub TE, Wheatley AK, Juno JA (2022). Neutralising antibody titres as predictors of protection against SARS-CoV-2 variants and the impact of boosting: a meta-analysis. Lancet Microbe.

[37] Khoury DS, Cromer D, Reynaldi A, Schlub TE, Wheatley AK, Juno JA (2021). Neutralizing antibody levels are highly predictive of immune protection from symptomatic SARS-CoV-2 infection. Nat Med.

[38] Fendler A, Shepherd STC, Au L, Wu M, Harvey R, Schmitt AM (2022). Omicron neutralising antibodies after third COVID-19 vaccine dose in patients with cancer. Lancet.

[39] Erdoğan AP, Ekinci F, Akçalı S, Göksel G (2022). Factors affecting the serologic response to SARS-CoV-2 vaccination in patients with solid tumors: a prospective study. J Infect Chemother.

[40] Lee E-J, Cines DB, Gernsheimer T, Kessler C, Michel M, Tarantino MD (2021). Thrombocytopenia following Pfizer and Moderna SARS-CoV-2 vaccination. Am J Hematol.

[41] Greinacher A, Thiele T, Warkentin TE, Weisser K, Kyrle PA, Eichinger S (2021). Thrombotic thrombocytopenia after ChAdOx1 nCov-19 vaccination. N. Engl J Med.

[42] Goddard K, Lewis N, Fireman B, Weintraub E, Shimabukuro T, Zerbo O, et al. Risk of myocarditis and pericarditis following BNT162b2 and mRNA-1273 COVID-19 vaccination. Vaccine. 2022;40:5153–9. 10.1016/j.vaccine.2022.07.007 PMC9273527 35902278

[43] Miyakis S, Lockshin MD, Atsumi T, Branch DW, Brey RL, Cervera R (2006). International consensus statement on an update of the classification criteria for definite antiphospholipid syndrome (APS). J Thrombosis Haemost.

[44] Borghi MO, Bombaci M, Bodio C, Lonati PA, Gobbini A, Lorenzo M (2022). Anti-phospholipid antibodies and coronavirus disease 2019: vaccination does not trigger early autoantibody production in healthcare workers. Front Immunol.

[45] Signorelli F, Balbi GGM, Aikawa NE, Silva CA, Kupa LDVK, Medeiros-Ribeiro AC (2022). Immunogenicity, safety, and antiphospholipid antibodies after SARS-CoV-2 vaccine in patients with primary antiphospholipid syndrome. Lupus.

[46] Mohan C, Adams S, Stanik V, Datta SK (1993). Nucleosome: a major immunogen for pathogenic autoantibody-inducing T cells of lupus. J Exp Med.

[47] Bingle L, Barnes FA, Lunn H, Musa M, Webster S, Douglas CWI (2009). Characterisation and expression of SPLUNC2, the human orthologue of rodent parotid secretory protein. Histochemistry Cell Biol.

[48] Epp TA, Dixon IMC, Wang H-Y, Sole MJ, Liew C-C (1993). Structural organization of the human cardiac α-myosin heavy chain gene (MYH6). Genomics.

[49] Lv H, Havari E, Pinto S, Gottumukkala RVSRK, Cornivelli L, Raddassi K (2011). Impaired thymic tolerance to α-myosin directs autoimmunity to the heart in mice and humans. The. J Clin Investig.

[50] Chang SE, Feng A, Meng W, Apostolidis SA, Mack E, Artandi M (2021). New-onset IgG autoantibodies in hospitalized patients with COVID-19. Nat Commun.

[51] Anand P, Lenehan PJ, Niesen M, Yoo U, Patwardhan D, Montorzi M (2022). Genetic alteration of human MYH6 is mimicked by SARS-CoV-2 polyprotein: mapping viral variants of cardiac interest. Cell Death Discov.

