# Supplementary material for: Longitudinal efficacy and toxicity of SARS-CoV-2 vaccination in cancer patients treated with immunotherapy
Source: Cell Death Dis. 2023 Jan 20;14(1):49. doi: 10.1038/s41419-022-05548-4 (PMC9853486; doi:10.1038/s41419-022-05548-4)
Supplement: Supplementary file 1 — Supplementary Tables and Figures [file 41419_2022_5548_MOESM1_ESM.docx]

**SUPPLEMENTARY FILE**

**(A)**

**(B)**

**Figure S1: VIVACIOUS study design and CONSORT diagram**

**(A),** Study design and analyses conducted; blood sampling timepoints at baseline (BASE, between 1^st^ and 2^nd^ dose (PRE2D), at 1 week (1W), 1 month (1M), 4-6 months (4-6M) and 10-12 months (10-12M) following the second vaccine dose **(B),** CONSORT diagram of patients received initial vaccination series and boosters, *patients who died of their malignant disease.

**Table S1. List of antigens used to generate antigen microarrays**

| **Antigen** | **Antigen full name** | **Company/source** | **Catalog number** |
| --- | --- | --- | --- |
| A1AR | Alpha 1A adrenergic receptor fragment | GenScript | custom peptide |
| Actin (Bovine) | Actin | Sigma | A3653 |
| Actin (Rabbit) | Actin (rabbit muscle) | Molecular Probes | A12375 |
| Aggrecan | Aggrecan | Sigma | A1960 |
| Aldolase, Type X | Aldolase, Type X | Sigma | A2714 |
| Alpha elastin | Alpha-elastin | Fred Keeley lab;University of Toronto | recombinant protein prepared in-house |
| alpha-Actinin | Alpha-actinin | Sigma | A9776 |
| Alpha-KGDH | Alpha-ketoglutarate dehydrogenase | Sigma | K1502 |
| alphaB-crystallin | Alpha B-crystallin | Enzo (Stressgen) | ADI-SPP-228 |
| Asparaginyl-tRNA Synthetase | Asparaginyl-tRNA Synthetase (KS) | Diarect | 30100 |
| AT1 | angiotensin 2 receptor (AT1R) fragment | GenScript | custom peptide |
| B1AR | Beta 1 adrenergic receptor (B1AR) fragment | GenScript | custom peptide |
| B2AR | Beta 2 adrenergic receptor (B2AR) fragment | GenScript | custom peptide |
| BCOADC-E2 | branched chain 2-oxo-acid dehydrogenase complex | Diarect | 17700 |
| Beta 2 Glyc | Beta 2 Glycoprotein I (recombinant human) | Diarect | 14900 |
| beta 2 GPI | Beta 2 Glycoprotein I (non-recombinant, Human) | Diarect | 11400 |
| beta 2 GPI | Beta 2 Glycoprotein I (non-recombinant, Human) | Diarect | 11300 |
| Bovin Histone H4 and H2A | Bovine Histone H4 and H2A | Immunovision | HIS-1002 |
| Bovin Histone subclass F1 | Bovine Histone subclass F1 | Immunovision | HIS-1001 |
| Bovine Histone H2b (F2b) | Bovine Histone H2b (F2b) | Immunovision | HIS-1003 |
| Bovine Histone H3 | Bovine Histone H3 | Immunovision | HIS-1004 |
| BPI | Bactericidal/Permeability Increasing protein | Diarect | 19200 |
| C1q (purified non-recombinant) | Complement component C1q | Sigma | C1740 |
| Carbonic Anhydrase | Carbonic Anhydrase VI (CAG) | MyBiosource | MBS2011329 |
| Cardiolipin C1649 | Cardiolipin | Sigma | C1649 |
| CENP-A | Centromere Protein A | Diarect | 16900 |
| CENP-B | Centromere Protein B | Diarect | 12500 |
| Collagen I | Collagen, I | Sigma | C7774 |
| Collagen III | Collagen, III | Sigma | C4407 |
| Collagen IV | Collagen, IV | Sigma | C5533 |
| **Antigen** | **Antigen full name** | **Company/source** | **Catalog number** |
| Collagen V | Collagen, V | Sigma | C3657 |
| Collagen VI | Collagen, VI | Sigma | C7521 |
| Desmin | Desmin | GenWay | GWB-2E8E72 |
| DNA Topoisomerase I (Scl-70; non recombinant; bovine) | DNA Topoisomerase I | Diarect | 11500 |
| dsDNA (genomic) | Deoxyribonucleic acid, double stranded | Sigma | D1626 |
| dsDNA (plasmid) | DNA circualar plasmid, Double Stranded | Diarect | 12300 |
| Ebna peptide | EBV nuclear antigen | Stanford | custom peptide |
| Enolase | Enolase | Sigma | E0379 |
| Fib I | Fibrinogen, type I | Sigma | F3879 |
| Fib I-S | Fibrinogen, type I-S | Sigma | F8630 |
| Fib IV | Fibrinogen, type IV | Sigma | F4753 |
| GBM | Glomerular Basement Membrane (GBM; dissociated) | Diarect | 16800 |
| Gliadin | Gliadin | Diarect | 19500 |
| Glycyl-tRNA Synthetase (EJ) | Glycyl-tRNA synthetase | Diarect | 11100 |
| GP2 | Zymogen granule membrane glycoprotein 2 | Diarect | 19600 |
| gp210 | glycoprotein 210 | Diarect | 19000 |
| Grp78/BiP | GRP78 (HSPa5 or BiP) immunoglobulin heavy chain-binding protein | Enzo (Stressgen) | ADI-SPP-765 |
| HCEC cytop | Human Cardiac Endothelial Cells Lysate | Dr. Vivek Rao's lab (UHN) | custom lysate prepared in-house |
| HCEC memb | Human Cardiac Endothelial Cell Lysate | Dr. Vivek Rao's lab (UHN) | custom lysate prepared in-house |
| HCEC total (SDS) | Human Cardiac Endothelial Cell Lysate | Dr. Vivek Rao's lab (UHN) | custom lysate prepared in-house |
| HCEC total (Triton) | Human Cardiac Endothelial Cell Lysate | Dr. Vivek Rao's lab (UHN) | custom lysate prepared in-house |
| Heparin | Heparin Sulfate | Sigma | H7640 |
| Histone H2A-H2B dimers | Histone H2A-H2B dimers | EMDMillipore | 14-1052 |
| Histone H2B | Histone H2B | Active Motif | 31492 |
| Histone H3 | Histone H3 (1-136 aa) | RayBiotech | 268-11222-1 |
| Histone H4 | Histone H4 (1-103 aa) | RayBiotech | 268-11223-1 |
| HMG CoA | 3-hydroxy-3-methylglutaryl-Coenzyme A Reductase | Cayman | 14944 |
| HSP 27 | Heat shock protein 27 | Enzo (Stressgen) | ADI-SPP-715 |
| HSP 40 | Heat shock protein 40 | Enzo (Stressgen) | ADI-SPP-400 |
| HSP 47 | Heat shock protein 47 | Enzo (Stressgen) | ADI-SPP-535 |
| HSP 60 | Heat shock protein 60 | Enzo (Stressgen) | ADI-NSP-540-E |
| HSP 70 | Heat shock protein 70 | Enzo (Stressgen) | ADI-ESP-555 |
| **Antigen** | **Antigen full name** | **Company/source** | **Catalog number** |
| HSP 90 | Heat shock protein 90 | Enzo (Stressgen) | ADI-SPP-770 |
| human albumin | Human albumin | Sigma | A9731 |
| human C1q | Native Human C1q protein | Abcam | ab96363 |
| Human core histones | Human core histones | RayBiotech | 268-11229-1 |
| human fgl2 | fibrinogen-like 2 | Dr. Gary Levy (UHN) | recombinant protein prepared in-house |
| Human H1 Chromatin | Human H1 Chromatin | Dr. Joan Wither (UHN) | prepared in-house |
| Human IgA | Human IgA | Jackson Immunoresearch | 009-000-011 |
| Human IgE | Human IgE | Abcam | ab65866 |
| Human IgG | Human IgG | Jackson Immunoresearch | 009-000-003 |
| human IgG F(ab)2 | Human IgG F(ab)2 fragment | Jackson Immunoresearch | 009-000-006 |
| human IgG Fc | Human IgG Fc fragment | Jackson Immunoresearch | 009-000-008 |
| Human IgM | Human IgM | Jackson Immunoresearch | 009-000-012 |
| human intestinal smooth muscle cell | Human Intestinal Smooth Muscle Cell Lysate | ScienCell Research Laboratories | 2916 |
| Human LEDGF | lens epithelium-derived growth factor) or dense fine speckles 70kD protein (DFS 70) | R&D Systems | 3468-LE-050 |
| Human LGALS3/Galectin 3 | Human LGALS3/Galectin 3 | LSBio | LS-G478 |
| human nucleosome | Human nucleosome | EpiGex | EPX-01-NCN |
| ICAM-1 | Inter-Cellular Adhesion Molecule 1 | ProSci | 96-400 |
| Insulin | Insulin | Sigma | I2643 |
| Intrinsic Factor | Intrinsic Factor | Diarect | 16700 |
| Jo-1 | Histidyl-tRNA synthetase | Diarect | 12900 |
| kidney endothelial cell lysate | Kidney Endothelial Cell Lysate | Dr. Anna Konvalinka (UHN) | custom lysate |
| Ku (p70/p80) | Ku (p70/p80) | Diarect | 17300 |
| La (SS-B) Antigens | La (SS-B) Antigens (47 kD ribonucleic protein) | Immunovision | SSB-3000 |
| La/SS-B (Recombinant, human diarect) | La (SS-B) Antigens (47 kD ribonucleic protein) | Diarect | 12800 |
| Laminin | Laminin | Sigma | L2020 |
| LCI | liver cytosol type 1 antigen | Diarect | 13700 |
| LG3 | immunogenic fragment of perlecan | Dr. Marie-Josee Hebert (University of Montreal) | recombinant protein prepared in-house |
| LKM 1 hp | Cytochrome p450 2D6 | Diarect | 19800 |
| M2 | The branched-chain α-ketoacid dehydrogenase complex: BCKDH complex) | Diarect | 18000 |
| **Antigen** | **Antigen full name** | **Company/source** | **Catalog number** |
| M2AR | M2 Muscarinc Receptor | GenScript | custom peptide |
| MDA5 | melanoma differentiation-associated protein 5 | Diarect | 30000 |
| Measles | Measles antigen | Meridian | R14120 |
| Mi-2 | Subunit of the nucleosome remodeling deacetylase (NuRD) complex | Diarect | 18100 |
| mouse fgl2 | Mouse fibrinogen-like 2 | Dr. Gary Levy (UHN) | recombinant protein prepared in-house |
| mouse IgG F(ab')2 | mouse IgG F(ab')2 | Jackson Immunoresearch | 015-000-006 |
| mouse IgG Fc | mouse IgG Fc | Jackson Immunoresearch | 015-000-008 |
| mouse IgM | mouse IgM | Biolegend | MM-30 |
| MPO | Myeloperoxidase (MPO) | Diarect | 18500 |
| Muscarinic Rceptor 3 | Muscarinic acetylcholine receptor 3, fragment | GenScript | custom peptide |
| MYH6 | alpha cardiac myosin heavy chain | CUSABIO | CSB-YP015299HU |
| Myosin Bind Protein C | Myosin Binding Protein C | Dr. Sadayappan (Loyola University) | recombinant protein prepared in-house |
| Myosin | Myosin, calcium activated | Sigma | M1636 |
| Nucleolin | Nucleolin | Diarect | 19700 |
| Nucleosome (non-recombinant; bovine) | Nucleosome | Diarect | 31000 |
| Nup62 | nucleoporin Nup62 | Diarect | A194 |
| OGDC-E2 | 2-oxoglutarate dehydrogenase complex | Diarect | 17800 |
| Ox LDL | Copper Oxidized human LDL | Academy Bio-Medical Company | 20P-OX-L102 |
| PBS | phosphate buffered saline | Wisent Bioproducts | 311-010-CL |
| PCNA | Proliferating cell nuclear antigen | Diarect | 36600 |
| PDC-E2 | E2 component of mitochondrial pyruvate dehydrogenase complex | Diarect | A179 |
| PDH | Pyruvate dehydrogenase | Sigma | P7032 |
| PL-12 | Alanyl-tRNA Synthase | Diarect | 15700 |
| PL-7 | Threonyl-tRNA Synthase | Diarect | 15600 |
| PM/Scl 100 | polymyositis/scleroderma (PM/Scl) complex 100 | Diarect | 16000 |
| PM/Scl 75 | polymyositis/scleroderma (PM/Scl) complex 75 | Diarect | 17000 |
| Porcine Myosin Heart | Myosin, calcium activated, porcine heart | Sigma | M0531 |
| PR3 | Proteinase 3 | Diarect | PR3 |
| Proteoglycan | Proteoglycan | Sigma | P5864 |
| Recombinant Histone H2A (hu) | Recombinant Histone H2A (hu) | Active Motif | 31490 |
| Ribo P0 | Ribosomal Phosphoprotein P0 | Diarect | 14100 |
| Ribo P1 | Ribosomal Phosphoprotein P1 | Diarect | 14200 |
| **Antigen** | **Antigen full name** | **Company/source** | **Catalog number** |
| Ribo P2 | Ribosomal Phosphoprotein P2 | Diarect | 14300 |
| RNP/Sm | RNP/Sm (non-recombinant; bovine) | Diarect | 11600 |
| Ro/SS-A | Ro/SS-A (52 kDa, human recombinant) | Diarect | 12700 |
| Ro/SS-A | Ro/SS-A (60 kDa, non-recombinant; bovine) | Diarect | 15500 |
| Ro/SS-A | Ro/SS-A (60 kDa, recombinan) | Diarect | 17400 |
| SCGB1A1 | Secretoglobin family 1A member 1 | Sigma | APrEST78092 |
| Scl-70 Full | DNA Topoisomerase I (Scl-70) full length | Diarect | 12400 |
| Scl-70: trunc | DNA Topoisomerase I (Scl-70) truncated | Diarect | 14500 |
| Sm Antigens | Sm (non-recombinant; bovine) | Diarect | 17500 |
| SmD | SmD | Diarect | 11700 |
| SmD1 | Human small nuclear ribonucleoprotein D1 polypeptide (SNRPD1; SmD1). | Diarect | 11800 |
| SmD2 | Human small nuclear ribonucleoprotein D2 polypeptide (SNRPD2; SmD2). | Diarect | 11900 |
| SmD3 | Human small nuclear ribonucleoprotein D3 polypeptide (SNRPD3; SmD3). | Diarect | 12000 |
| snRNP 68 | U1-snRNP 68 Protein | Diarect | 13000 |
| snRNP 68 B/B | U1-snRNP 68 Protein B/B | Diarect | 13300 |
| snRNP A | U1-snRNP A Protein | Diarect | 13100 |
| snRNP C | U1-snRNP C Protein | Diarect | 13200 |
| SP-D | Surfactant protein D | Novusbio | 1920-SP |
| Sp100 | Sp100 nuclear antigen (Speckled 100 kDa) | Diarect | 18900 |
| SPLUNC2 | Human Parotid Secretory Protein (SPLUNC2: Recombinant Short Palate, Lung And Nasal Epithelium Carcinoma Associated Protein 2) | MyBiosource | MBS2122495 |
| SRP54 | signal recognition particle (SRP) 54 GTPase protein | Diarect | 18400 |
| ssDNA | Deoxyribonucleic acid, single stranded | Sigma | D8899 |
| thyroglobulin #2 | dimeric glycoprotein thyroglobulin | Diarect | A12200 |
| Thyrogobulin | dimeric glycoprotein thyroglobulin | Diarect | A12200 |
| TIF1 gamma | transcription intermediary factor 1-gamma | Diarect | 11000 |
| TPO | Thyroid peroxidase | Diarect | 12100 |
| Tropoelastin | Tropoelastin | Fred Keeley lab (Uni of Toronto) | recombinant protein prepared in-house |
| Tropomyosin | Tropomyosin | Sigma | T2400 |
| Troponin C | Troponin C | CalBioreagents | A093 |
| Troponin I | Troponin I | Sigma | T9924 |
| Troponin T | Troponin T | Sigma | T0175 |
| tTG baculovirus | Tissue transglutaminase baculovirus | Diarect | 15200 |
| **Antigen** | **Antigen full name** | **Company/source** | **Catalog number** |
| tTG E. coli | Tissue transglutaminase E. coli | Diarect | 14400 |
| TUBA1B protein | Tubulin alpha-1B chain | Abnova | H00010376-P01 |
| Vimentin | Vimentin | Cedarlane | CLPR0309 |
| whole histones | Whole Histones | Immunovision | HIS-1000 |
| whole mouse IgG | mouse IgG | Jackson immunoresearch | [015-000-003](https://www.jacksonimmuno.com/catalog/products/015-000-003) |

**Figure S2: Antibody level and neutralization at 1M and change after 1M.**

**(A),** Anti-RBD antibody levels at 1M following completion of initial vaccination series between non-immunotherapy (non-IO, cyan, n=15) and immunotherapy groups (IO, magenta, n=33). **(B),** Neutralizing antibody levels at 1M following completion of initial vaccination series between non-immunotherapy (non-IO, cyan, n=15) and immunotherapy groups (IO, magenta, n=33). Mann-Whitney tests were used for comparison. **(C),** change in levels of anti-RBD antibody (left panel) and neutralizing antibody (right panel) in relation to time after completion of initial vaccination series in patients who had 2 vaccine doses only; colours indicate vaccine formulation for initial vaccination series (navy blue: AstraZeneca, red: AstraZeneca followed by Pfizer, green: Moderna, purple: Pfizer followed by Moderna and light green: Pfizer). Circled level belongs to patient who contracted SARS-CoV-2 at 228 days after receiving the second dose and has not received any booster vaccine doses.

**Table S2: Univariate regression models of log-transformed antibody levels and neutralization levels by various predictors.**

|  | **Antibody levels at 1M following 2 vaccination doses** | | |
| --- | --- | --- | --- |
|  | **Estimate (95%CI)** | **p-value** | **N** |
| **Age** | -0.03 (-0.08, 0.01) | 0.93 | 48 |
| **Sex** |  | **0.01** | 48 |
| Female | Reference |  | 22 |
| Male | -1.62 (-2.55, -0.69) |  | 26 |
| **Therapy** |  | 1 | 48 |
| IO | Reference |  | 33 |
| Non-IO | -0.19 (-1.31, 0.93) |  | 15 |
| **Chemotherapy** |  | 0.1 | 48 |
| No | Reference |  | 42 |
| Yes | -1.93 (-3.39, -0.46) |  | 6 |
| **time since first dose** | -4.4e-06 (-0.02, 0.02) | 1 | 48 |
| **Vaccine Type** |  |  | 48 |
| P+P | Reference |  | 33 |
| AZ+AZ | -1.83 (-3.96, 0.31) | 0.73 | 3 |
| AZ+P | 0.13 (-3.46, 3.73) | 1 | 1 |
| M+M | -0.18 (-1.65, 1.30) | 1 | 7 |
| P+M | 1.07 (-0.81, 2.94) | 1 | 4 |
|  | **Neutralization levels at 1M following 2 vaccination doses** | | |
|  | **Estimate(95%CI)** | **p-value** | **N** |
| **Age** | -0.26 (-0.75, 0.23) | 1 | 48 |
| **Sex** |  | **0.03** | 48 |
| Female | Reference |  | 22 |
| Male | -16.38 (-26.56, -6.20) |  | 26 |
| **Therapy** |  | 1 | 48 |
| IO | Reference |  | 33 |
| Non-IO | -1.41 (-13.53, 10.71) |  | 15 |
| **Chemotherapy** |  | 1 | 48 |
| No | Reference |  | 42 |
| Yes | -13.18 (-29.72, 3.35) |  | 6 |
| **time since first dose** | -0.23 (-0.48, 0.02) | 0.75 | 48 |
| **Vaccine Type** |  |  | 48 |
| P+P | Reference |  | 33 |
| AZ+AZ | -21.42 (-44.55, 1.71) | 0.75 | 3 |
| AZ+P | 9.25 (-29.68, 48.18) | 1 | 1 |
| M+M | 2.34 (-13.62, 18.30) | 1 | 7 |
| P+M | 6.42 (13.89, 26.73) | 1 | 4 |

**Abbreviations**: P+P: Pfizer followed by Pfizer; AZ+AZ: AstraZeneca followed by AstraZeneca; AZ+P: AstraZeneca followed by Pfizer; M+M: Moderna followed by Moderna, P+M: Pfizer followed by Moderna.

**Figure S3: Prevalence of immune-related adverse events, relative to time of vaccination dose.**

**Table S3: Immune-related adverse events**

| **Patient ID** | **AE severity** | **Vaccine (1st dose)** | **D1 to AE days** | **Adverse event** |
| --- | --- | --- | --- | --- |
| **After 1st dose of vaccine** | | | | |
| LIB-26-0013 | 3 | Moderna | 12 | Increased lipase |
| LIB-26-0043 | 2 | Pfizer | 21 | Hypothyroidism |
| **After 2nd dose of vaccine** | | | | |
| LIB-26-0009 | 2 | Pfizer | 64 | Skin hypopigmentation |
| LIB-26-0013 | 2 | Moderna | 1 | Neck lymphadenopathy |
| LIB-26-0013 | 2 | Moderna | 1 | Fatigue |
| LIB-26-0015 | 2 | Pfizer | 73 | Adrenal insufficiency |
| LIB-26-0020 | 2 | Moderna | 80 | Diarrhoea |
| LIB-26-0027 | 2 | Pfizer | 57 | Splenomegaly |
| LIB-26-0029 | 2 | Moderna | 39 | Adrenal insufficiency |
| LIB-26-0033 | 2 | Pfizer | 38 | Diarrhoea |
| LIB-26-0038 | 2 | Moderna | 8 | Rash |
| **After 3rd dose of vaccine** | | | | |
| LIB-26-0031 | 2 | Pfizer | 89 | Hypothyroidism |
| LIB-26-0040 | 2 | Pfizer | 29 | Transaminitis |
| **After 4th dose of vaccine** | | | | |
| LIB-26-0024 | 2 | Pfizer | 38 | Rash |
| LIB-26-0024 | 2 | Pfizer | 84 | Pruritus |
| LIB-26-0031 | 2 | Pfizer | 20 | Rash |


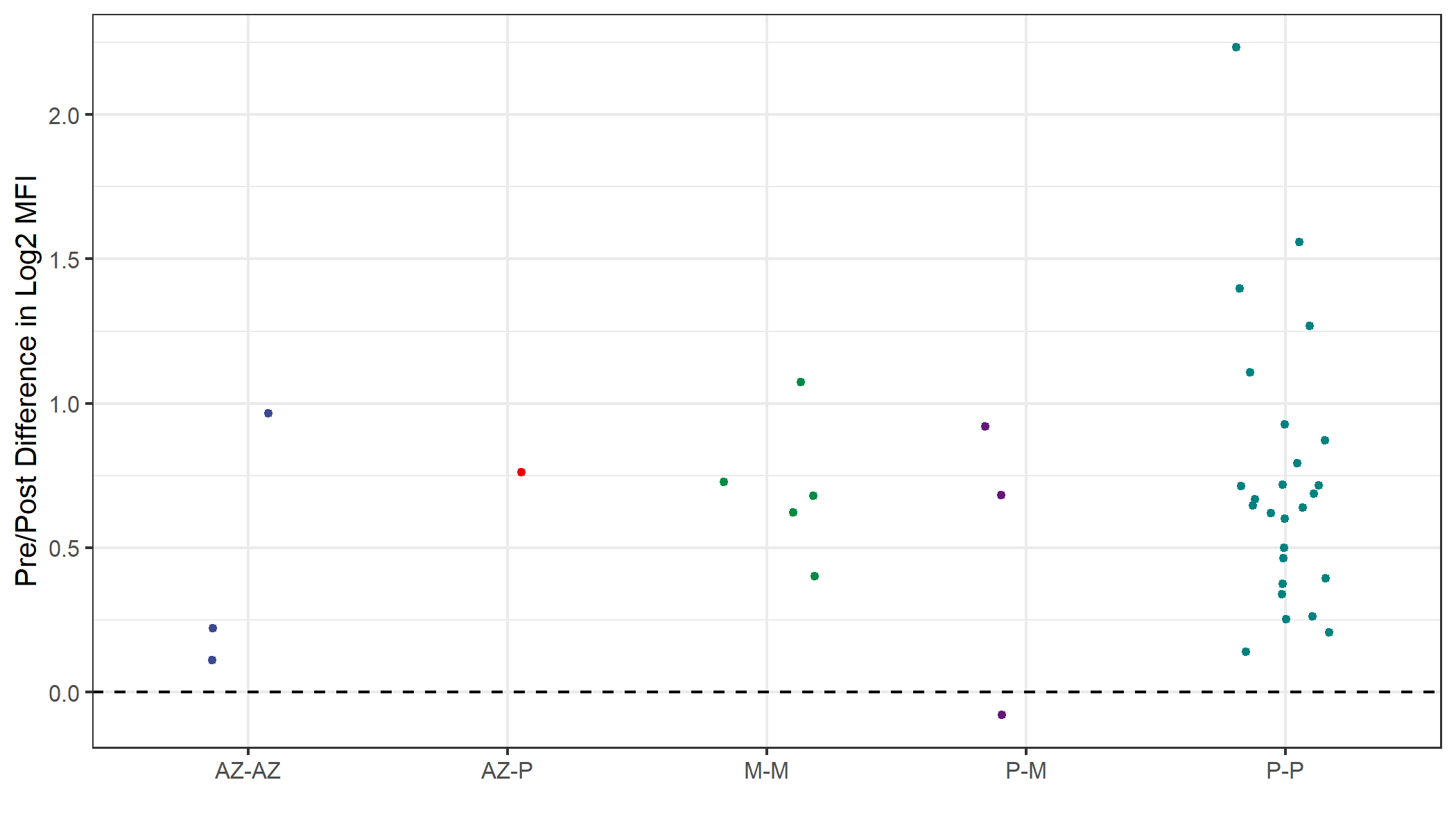


**Figure S4: IgG MHY6 by vaccine type**

Plot of mean differences of IgG MHY6 auto-antibody levels pre and post vaccination (following second vaccination), as measured by median log2 fluorescence intensity (MFI) with a 161 self-antigen array. Abbreviations: P+P: Pfizer followed by Pfizer; AZ+AZ: AstraZeneca followed by AstraZeneca; AZ+P: AstraZeneca followed by Pfizer; M+M: Moderna followed by Moderna, P+M: Pfizer followed by Moderna.


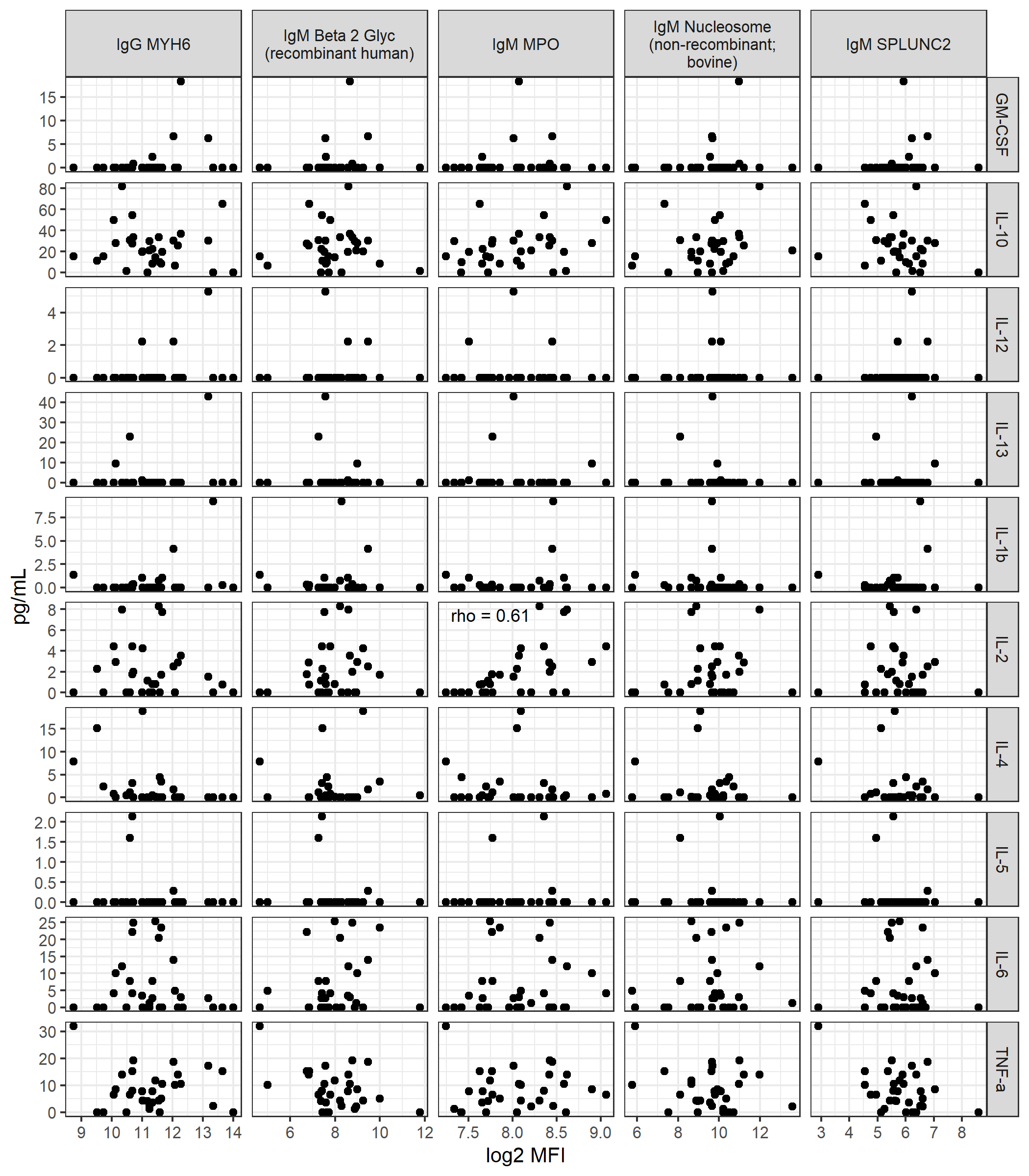


## Figure S5: Correlation between cytokine levels and autoantibodies post 2^nd^ vaccine dose.

Spearman’s correlation was used to test the association between cytokine levels and auto-antibody levels at 1 week post 2^nd^ vaccination dose in a cohort of IO-treated patients (n=33) and non-IO treated patient samples (n=5).

| **Antibody levels at one month** | n=48 |
| --- | --- |
| **sVNA** |  |
| Mean (sd) | 85.3 (19.1) |
| Median (Min,Max) | 94.0 (11.5, 96.6) |
| **Roche** |  |
| Mean (sd) | 5826.1 (20932.9) |
| Median (Min,Max) | 1214.5 (5.5, 145200.0) |
|  |  |
| **Post vaccination auto-antibody levels** | n=38 |
| **IgG MYH6** |  |
| Mean (sd) | 11.5 (1.2) |
| Median (Min,Max) | 11.3 (8.7, 14.0) |
| **IgM Beta 2 Glyc (recombinant human)** |  |
| Mean (sd) | 8.0 (1.3) |
| Median (Min,Max) | 7.9 (4.6, 11.8) |
| **IgM MPO** |  |
| Mean (sd) | 8.0 (0.5) |
| Median (Min,Max) | 7.9 (7.2, 9.1) |
| **IgM Nucleosome (non-recombinant; bovine)** |  |
| Mean (sd) | 9.6 (1.6) |
| Median (Min,Max) | 9.7 (5.8, 13.6) |
| **IgM SPLUNC2** |  |
| Mean (sd) | 5.8 (1.0) |
| Median (Min,Max) | 5.8 (2.9, 8.6) |
|  |  |
| **Cytokine levels at 1 week** | n=42 |
| **GM-CSF** |  |
| Mean (sd) | 0.8 (3.1) |
| Median (Min,Max) | 0.0 (0.0, 18.3) |
| Missing | 1 |
| **IL-1b** |  |
| Mean (sd) | 0.5 (1.6) |
| Median (Min,Max) | 0.0 (0.0, 9.2) |
| Missing | 1 |
| **IL-2** |  |
| Mean (sd) | 1.9 (2.6) |
| Median (Min,Max) | 0.8 (0.0, 9.7) |
| Missing | 1 |
| **IL-4** |  |
| Mean (sd) | 2.6 (4.8) |
| Median (Min,Max) | 0.0 (0.0, 18.9) |
| Missing | 5 |
| **IL-5** |  |
| Mean (sd) | 0.3 (1.1) |
| Median (Min,Max) | 0.0 (0.0, 6.5) |
| **IL-6** |  |
| Mean (sd) | 14.2 (50.8) |
| Median (Min,Max) | 2.8 (0.0, 329.7) |
| **Cytokine levels at 1 week** | n=42 |
| **IL-10** |  |
| Mean (sd) | 25.4 (23.6) |
| Median (Min,Max) | 21.2 (0.0, 124.5) |
| Missing | 1 |
| **IL-12** |  |
| Mean (sd) | 0.4 (1.4) |
| Median (Min,Max) | 0.0 (0.0, 6.9) |
| **IL-13** |  |
| Mean (sd) | 1.9 (7.6) |
| Median (Min,Max) | 0.0 (0.0, 42.9) |
| Missing | 1 |
| **TNF-a** |  |
| Mean (sd) | 8.8 (7.7) |
| Median (Min,Max) | 7.9 (0.0, 31.9) |
| Missing | 1 |

## Table S3: variability between groups of antibodies, autoantibodies and chemokines presented.
